# Iron biofortification and availability in the mycelial biomass of edible and medicinal basidiomycetes cultivated in sugarcane molasses

**DOI:** 10.1038/s41598-020-69699-0

**Published:** 2020-07-30

**Authors:** Simone Schenkel Scheid, Maria Graciela Iecher Faria, Leonardo Garcia Velasquez, Juliana Silveira do Valle, Affonso Celso Gonçalves, Douglas Cardoso Dragunski, Nelson Barros Colauto, Giani Andrea Linde

**Affiliations:** 1 Graduate Program of Biotechnology Applied to Agriculture, Universidade Paranaense, Mascarenhas de Moraes, 4282, Umuarama, PR 87502-210 Brazil; 2 Medicinal Plants and Herbal Remedies in Basic Care, Universidade Paranaense, Umuarama, PR, Brazil; 3 Laboratory of Environmental Chemistry, Centre of Agricultural Science, West Paraná State University, Marechal Cândido Rondon, PR Brazil; 4 Center of Engineering and Exact Sciences, West Paraná State University, Toledo, PR Brazil

**Keywords:** Environmental microbiology, Microbiology techniques

## Abstract

Basidiomycetes can bioaccumulate high iron contents, but there are few studies on iron availability from the mycelial biomass in order to support their use as an iron-enriched fungal food. This study aimed to evaluate the in vitro iron bioaccumulation and availability in the mycelial biomass of edible and medicinal basidiomycetes grown in two distinct culture media. *Lentinus crinitus*, *Ganoderma lucidum*, *Schizophyllum commune*, *Pleurotus ostreatus*, *Pleurotus eryngii*, *Lentinula edodes*, and *Agaricus subrufescens* were grown in liquid culture medium of malt extract or sugarcane molasses to obtain iron-bioaccumulated mycelial biomass. *P. ostreatus* was the fungus that most bioaccumulated iron, followed by *S. commune*, and *P. eryngii*; they also had the highest mycelial biomass growth and iron transfer from the culture medium to the mycelial biomass. Mycelial iron availability is species-specific, regardless of the culture medium and the iron bioaccumulation capacity of the fungus in the mycelial biomass. Mycelial biomass of *S. commune*, followed by *G. lucidum*, *P. ostreatus*, and *P. eryngii*, associated with molasses culture medium, are the best choice for the production of iron-enriched mycelial biomass.

## Introduction

Iron is an essential metal for animal metabolism as it is involved in DNA synthesis, hemoglobin, and redox reactions^1^. The most recent report from the World Health Organization (WHO) estimated that around 1.62 billion people, mainly children and women, had iron deficiency anemia^2^ and considered it a global public health problem of epidemic proportions^3^. Besides anemia, iron deficiency causes other abnormalities such as deficiency of vitamin B12 and vitamin A, parasitic infections, chronic inflammation, and inherited disorders^4^. Moreover, in 2012, the WHO established a global nutrition target for 2025: “achieving a 50% reduction of anemia in women of reproductive age”^5^. The conventional medical treatment to anemia is iron supplementation, mainly ferrous sulfate, which is a strong pro-oxidant that has been related to chronic diseases such as cirrhosis, cardiovascular diseases, type-2 diabetes, and cancer when in excess^6,7^. Therefore, the conventional treatment of anemia may cause a health issue, and iron food fortification seems to be a favorable risk–benefit ratio to improve health and prevent some diseases.

Basidiomycetes can bioaccumulate metals of nutritional and pharmacological importance such as iron^8–10^, zinc^10,11^, selenium^12^, and lithium^13,14^. Vegetative mycelial biomass of some basidiomycetes are reported to present iron content of 3,616 mg kg^−1^ in *Pleurotus ostreatus*^8^ and 2,595 mg kg^−1^ in *Agaricus subrufescens*^10^. Therefore, the mycelial biomass of edible and medicinal basidiomycetes could be a source of several metals, such as iron, besides natural bioactive molecules. For instance, in order to meet an adult woman’s daily need of 18 mg iron^15^, an intake of 5-g mycelial-bioaccumulated iron (3,616 mg kg^−1^ iron in mycelial biomass) would be sufficient^8^. However, these authors did not consider the iron availability in the mycelial biomass. Determining the iron availability is important to indicate the metal-absorption capacity by an organism, according to the iron solubility level^2,3,6^. Yokota and coworkers^9^ had determined the in vitro iron availability in *P. ostreatus* basidiocarps; however, they did not study the metal bioaccumulation in the mycelial biomass that is capable of higher metal-accumulation than the basidiocarp^8,9^. Thus, the aim of this study was to evaluate the in vitro iron bioaccumulation and availability in the mycelial biomass of edible and medicinal basidiomycetes grown in two distinct culture media.

## Results

The two-way variance analysis showed significant effects of species and culture-medium on mycelial biomass growth, iron content in the mycelial biomass, iron transfer from culture medium to the mycelial biomass, solubilized iron from the mycelial biomass, iron availability from the mycelial biomass, and overall iron yield (Table 1).

**Table 1:** Effects of two-way analysis of variance (ANOVA).

Effect	F	df	P
**Mycelial biomass growth**			
Species	382.0	6.42	< 0.001
Culture-medium	204.0	1.42	< 0.001
Species × culture-medium	143.2	6.42	< 0.001
**Iron content in the mycelial biomass**			
Species	108.3	6.42	< 0.001
Culture-medium	2,126.3	1.42	< 0.001
Species × culture-medium	102.4	6.42	< 0.001
**Iron transfer from the culture medium to the mycelial biomass**			
Species	177.5	6.42	< 0.001
Culture-medium	1675.4	1.42	< 0.001
Species × culture-medium	150.0	6.42	< 0.001
**Solubilized iron from the mycelial biomass**			
Species	70.8	6.42	< 0.001
Culture-medium	1,464.0	1.42	< 0.001
Species × culture-medium	50.5	6.42	< 0.001
**Iron availability from the mycelial biomass**			
Species	468.4	6.42	< 0.001
Culture-medium	0.82	1.42	0.370
Species × culture-medium	2.0	6.42	0.076
**Overall iron yield**			
Species	138.9	6.42	< 0.001
Culture-medium	1,198.6	1.42	< 0.001
Species × culture-medium	92.6	6.42	< 0.001

### Mycelial biomass cultivation

**Figure 1:**
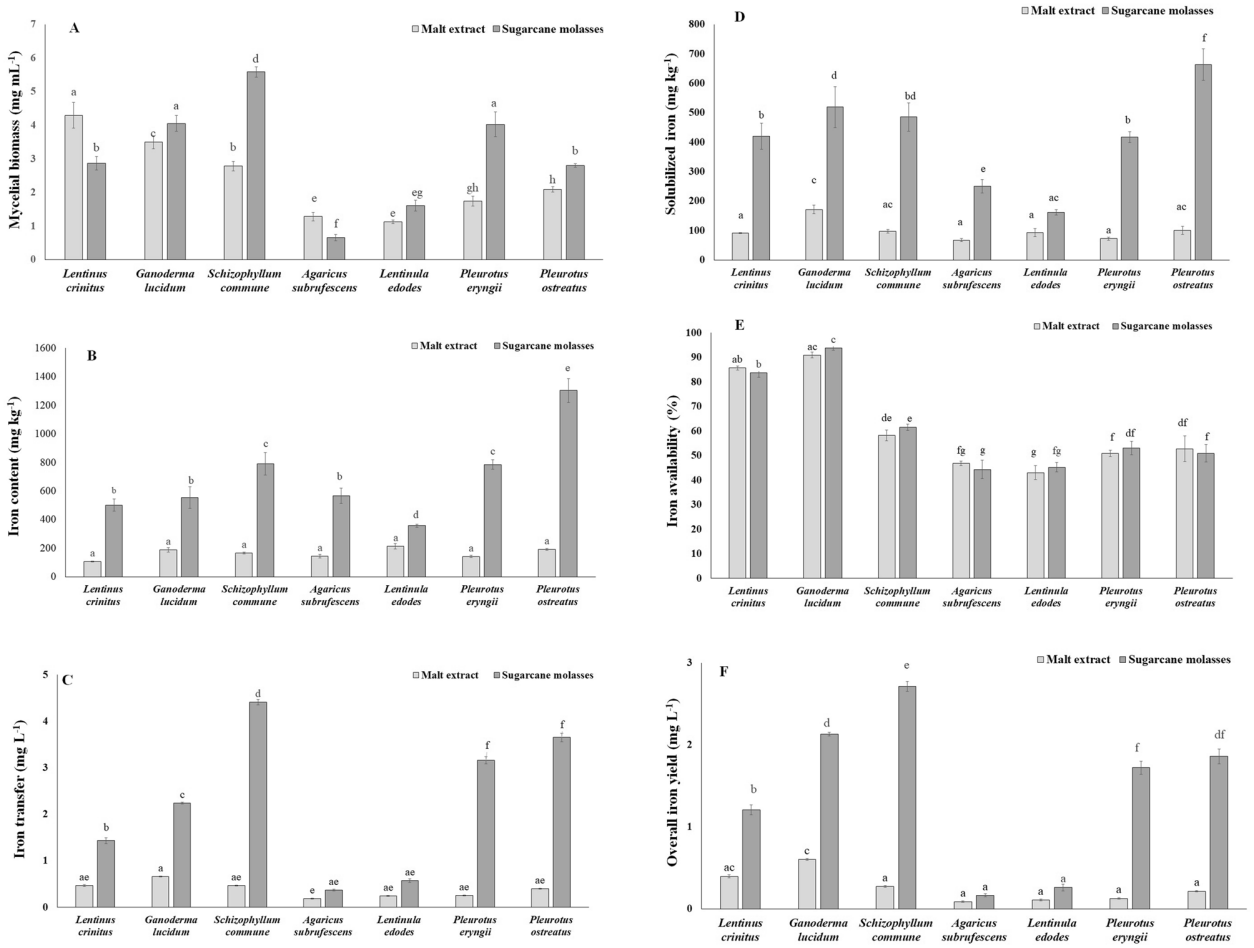
Mycelial biomass concentration (**A**), iron content in the mycelial biomass (**B**), iron transfer from culture medium to the mycelial biomass (**C**), solubilized iron from the mycelial biomass (**D**), iron availability from the mycelium biomass (**E**), and overall iron yield (soluble iron amount per liter of culture medium) (**F**) of basidiomycetes grown in liquid culture medium of malt-extract or sugarcane molasses for 21 days. Malt-extract culture medium: 60.00 mg L^−1^ nitrogen, 19.14 g L^−1^ carbohydrate, 0.116 mg L^−1^ iron, and 260.00 µg L^−1^ magnesium; sugarcane molasses culture medium: 120.00 mg L^−1^ nitrogen, 16.72 g L^−1^ carbohydrate, 91.23 mg L^−1^ iron, and 903.12 µg L^−1^ magnesium. Different letters indicate differences among data by Tukey's HSD test (*p* ≤ 0.05).

The mycelial biomass growth in culture medium of malt-extract ranged from 1.1 to 4.3 mg mL^−1^, and in sugarcane-molasses varied from 0.6 to 5.6 mg mL^−1^ (Fig. 1A). A two-way analysis of variance (ANOVA) showed significant differences between species and between culture-medium in biomass production (Table 1). Significant interaction of factors (species × culture-medium) was also registered due to the opposite effect of the culture-medium on the mycelial biomass of certain species. The highest (*p* ≤ 0.05) mycelial biomass growth was for *S. commune* in sugarcane-molasses culture medium (Fig. 1A). *A. subrufescens* and *L. crinitus* had mycelial biomass growth reduced by 1.5 and 1.97-fold, respectively, when cultivated in sugarcane-molasses culture medium. However, *G. lucidum*, *S. commune*, *P. ostreatus*, *P. eryngii*, and *L. edodes* mycelial biomass growth increased in sugarcane-molasses culture medium by 1.16, 2.00, 1.33, 2.31, and 1.42-fold, respectively, compared with the growth in malt-extract culture medium (Supplementary Information 1).

### Iron content in the mycelial biomass

The iron content of the mycelial biomass grown in culture medium of malt-extract ranged from 106 to 213 mg kg^−1^, and sugarcane-molasses ranged from 358 to 1,304 mg kg^−1^ (Fig. 1B). The iron content in the biomass on molasses were from 1.68 to 6.84-fold higher than the ones on malt-extract. Two-way ANOVA showed significant (*p* < 0.001) increase of iron content in the mycelial biomass on molasses compared to malt-extract for all fungi, and significant (*p* < 0.001) differences between species were also revealed (Table 1). The lowest (*p* ≤ 0.05) values for iron in the mycelial biomass were for *L. edodes*, followed by *L. crinitus*, *P. eryngii*, and *A. subrufescens* (Fig. 1B). The highest (*p* ≤ 0.05) iron content was 1,304 mg kg^−1^ for *P. ostreatus* on molasses, and this value was 2.9, 4.4, 5.4, and 12.5-fold higher than spinach, asparagus, shiitake, and broccoli, respectively (Table 2). In general, all fungi on molasses had greater iron content than vegetables (Fig. 1B, Table 2).

**Table 2:** Iron content and availability of vegetables and shiitake basidiocarp.

Vegetable/mushroom (scientific name)	Iron content (mg kg^−1^)	Solubilized iron (mg kg^−1^)	Iron availability (%)
Asparagus (*Asparagus officinalis*)	293.63 ± 38.24^b^	142.37 ± 5.73^b^	48.48 ± 5.19^b^
Broccoli (*Brassica oleracea*)	104.08 ± 6.75^c^	26.03 ± 1.54^c^	25.01 ± 2.48^c^
Spinach (*Spinacia oleracea*)	449.27 ± 53.11^a^	235.6 ± 9.28^a^	52.44 ± 7.84^a^
Shiitake (*Lentinula edodes*)	239.92 ± 24.37^b^	128.26 ± 10.16^b^	53.46 ± 4.10^a^

Different letters indicate statistical differences among data in the same row by Tukey's HSD test (*p* ≤ 0.05).

### Iron transfer from the culture medium to the mycelial biomass

A two-way ANOVA showed significant (*p* < 0.001) increase of iron transfer from the culture-medium to the mycelial biomass on molasses compared to malt-extract for all fungi, and significant (*p* < 0.001) differences between species were also revealed (Table 1). The iron transfer was from 2.05 and 12.64-fold higher for all fungi cultivated in the molasses culture medium. The iron transfer from the culture-medium to the mycelial biomass takes into consideration the mycelial biomass production as well as the iron content in the mycelial biomass. The highest value was for *S. commune*, followed by *P. ostreatus*, and *P. eryngii* (Fig. 1C). Although *S. commune* did not have the highest iron content in the mycelial biomass (Fig. 1B), it had great mycelial biomass growth on molasses (Fig. 1A). Thus, *S. commune* on molasses was the most efficient to transfer iron from the culture medium to the mycelial biomass.

### Solubilized iron from the mycelial biomass

A two-way ANOVA showed significant (*p* < 0.001) increase on iron solubilized for mycelial biomass on molasses compared to malt-extract for all fungi, and significant (*p* < 0.001) differences between species were also revealed (Table 1). The highest (*p* ≤ 0.05) solubilized iron value was 663 mg kg^−1^ for *P. ostreatus* on molasses, and the lowest (*p* ≤ 0.05) one was 161 mg kg^−1^ for *L. edodes* (Fig. 1D). The solubilized iron from the mycelial biomass cultivated on molasses ranged from 1.76 to 6.57-fold higher than the ones cultivated on malt-extract. The highest solubilized iron (1,304 mg kg^−1^ iron) from fungi was 2.8, 4.7, 5.2, and 25.5-fold higher than spinach, asparagus, shiitake, and broccoli, respectively (Table 2).

### Iron availability from the mycelial biomass

A two-way ANOVA showed significant (*p* < 0.001) increase of iron availability just between species (*p* < 0.001) (Table 1). Iron availability of the mycelial biomass grown in malt-extract or molasses was similar and ranged from 43 to 91%, and from 44 to 94%, respectively. The greatest (*p* ≤ 0.05) iron availability values were for *G. lucidum* and the lowest (*p* ≤ 0.05) ones for *L. edodes* and *A. subrufescens* (Fig. 1E). The iron availability is a species-specific fungal feature, regardless of the culture medium and the fungal capacity of iron bioaccumulation in the mycelial biomass (Fig. 1E). The iron availability from *G. lucidum* mycelial biomass was 1.7, 1.8, 1.9, and 3.7-fold higher than shiitake, spinach, asparagus, and broccoli, respectively (Table 2).

### Overall iron yield

The overall iron yield was obtained by multiplying the mycelial biomass, iron content in the mycelial biomass, and iron availability from the mycelial biomass for each fungal strain. This yield provides information on which fungus has the highest amount of soluble iron per liter of culture medium, which is an important parameter for the production of iron-enriched mycelial biomass. A two-way ANOVA showed significant (*p* < 0.001) increase of overall iron yield in the mycelial biomass on molasses compared to malt-extract for all fungi, and significant (*p* < 0.001) differences between species were also revealed (Table 1). The mycelial biomass of *S. commune*, followed by *G. lucidum*, *P. ostreatus*, and *P. eryngii*, cultivated on molasses had higher (*p* ≤ 0.05) capacity to transfer and solubilize iron (Fig. 1F). These fungal strains cultivated on molasses are the best choice for the production of iron-enriched mycelial biomass.

## Discussion

The molasses culture medium was the best option to produce iron-enriched mycelial biomass because it had high iron bioaccumulation in the mycelial biomass, and high mycelial biomass growth. Molasses is a viscous liquid byproduct from sugar production containing predominantly hexose, sucrose, and proteins^16^. It is estimated that, in 2019, Brazil produced 622 million tons of sugarcane (*Saccharum officinarum* L.) generating about 37 million tons of molasses^17^. Molasses is sold at US $60/ton and could be an alternative to malt extract sold at US $149–298/ton^16^ to produce iron-enriched mycelial biomass from nutraceutical basidiomycetes.

In our study, sugarcane molasses medium increased the iron content in the mycelial biomass from 1.7 to 6.8-folds; this may be related to the high iron content usually found in molasses that was 787-fold higher than in malt-extract. Almeida and coworkers^8^ showed—through a central composite design—that the iron content in the culture medium was the most important factor for the iron bioaccumulation in *P. ostreatus* mycelial biomass. The increase in the iron bioaccumulation is related to the high iron content in the culture medium for *A. subrufescens*^10^ and *P. ostreatus*^18^ mycelial biomass, and *P. ostreatus* basidiocarp^9^. Fungi have several strategies to bind iron from the culture medium such as acidification of the culture medium, conversion of ferric to ferrous iron, and secretion of iron chelating molecules as hydroxamates^19^. Thus, iron content in the culture medium is the most relevant factor for ion bioaccumulation in the mycelial biomass of basidiomycetes.

There are few reports on the in vitro iron availability for basidiomycetes. For Yokota et al.^9^, in vitro hydrolysis of *P. ostreatus* basidiocarp solubilized iron was 161.2 mg kg^−1^ iron. This value is fourfold lower than the same *P. ostreatus* strain used in our study (663.4 mg kg^−1^ iron). We also verified that *G. lucidum* mycelial biomass presented higher solubilized iron than the other basidiomycetes. Haneef et al.^20^ reported that the *P. ostreatus* hyphae diameter is larger than the *G. lucidum* one. This suggests that *G. lucidum* mycelial biomass has a greater contact area in the in vitro hydrolysis process, which could improve iron solubilization during in vitro hydrolysis. Moreover, Haneef et al.^20^ verified that *G. lucidum* showed a greater amount of lipids, whereas *P. ostreatus* mycelial biomass showed relatively more polysaccharides and chitin. Polysaccharides are macromolecules that physically and chemically can bind iron, reducing its solubility during in vitro hydrolysis. Kim et al.^21^ tested the production of meat analogues utilizing *A. bisporus* mycelium with better umami taste and textural properties compared to meat analogues of soy protein. This indicates that most basidiomycetes, considered health foods, can be used to bioaccumulate iron in the mycelial biomass as an alternative for the production of iron-enriched functional foods, including meat analogues. In addition, sugarcane molasses, a sugar-crystallization byproduct, is an economic alternative to produce biotechnological biofortified food.

## Conclusion

The fungi that most bioaccumulated iron are *P. ostreatus,* followed by *S. commune*, and *P. eryngii*; they also have the greatest mycelial biomass growth and iron transfer from the culture medium to the mycelial biomass. The mycelial iron availability is species-specific, regardless of the culture medium and the iron bioaccumulation capacity of the fungus in the mycelial biomass. The mycelial biomass of *S. commune*, followed by *G. lucidum*, *P. ostreatus*, and *P. eryngii*, associated with molasses culture medium, are the best choice for the production of iron-enriched mycelial biomass.

## Methods

### Biological material

*Lentinus crinitus* (L.) Fr. (U9-1), *Ganoderma lucidum* (Curtis) P.Karst. (U12-6), *Schizophyllum commune* Fr. (U6-7), *Pleurotus ostreatus* (Jacq.) P.Kumm. (U2-9), *Pleurotus eryngii* (DC.) Quél. (U12-5), *Lentinula edodes* (Berk.) Pegler (U6-1), and *Agaricus subrufescens* Peck (U7-1), also referred to as *Agaricus blazei* Murrill or *Agaricus brasiliensis* Wasser et al*.*, were from the culture collection of the Molecular Biology Laboratory of Paranaense University. All strains were registered in the National System of Genetic Patrimony Management and Associated Traditional Knowledge (SisGen, its acronym in Portuguese) under the number A04E776. The mycelia were grown in 20 g L^−1^ malt-extract agar (MEA), previously autoclaved at 121 °C for 20 min, in the dark, at 28 ± 1 °C. Homogenous mycelia without sectoring from the colony edge were used as inoculum for the assays.

### Mycelial biomass cultivation

The mycelial biomass has grown in two distinct liquid culture media: malt-extract and sugarcane molasses. Malt-extract is a standard culture medium for basidiomycetes, and sugarcane molasses is a byproduct of the sugar and ethanol industry, commonly used for the mycelial biomass growth of basidiomycetes to produce biomolecules and/or enzymes^22,23^. In an Erlenmeyer flask (250 mL), 150 mL culture medium at 20 g L^−1^ malt-extract or sugarcane molasses was autoclaved at 121 °C for 20 min. The malt-extract culture medium consisted of 60.00 ± 4.18 mg L^−1^ nitrogen, 19.14 ± 5.16 g L^−1^ carbohydrate, 0.116 ± 0.01 mg L^−1^ iron, and 260.00 ± 0.28 µg L^−1^ magnesium. The sugarcane-molasses culture medium consisted of 120.00 ± 1.85 mg L^−1^ nitrogen, 16.72 ± 0.48 g L^−1^ carbohydrate, 91.23 ± 0.001 mg L^−1^ iron, and 903.12 ± 0.001 µg L^−1^ magnesium. Each Erlenmeyer flask was inoculated with three 0.5 mm diameter MEA disks containing mycelia. The mycelial biomass has grown at 28 ± 1 °C for 21 days, in the dark, without agitation, and separated by centrifugation at 2,900g for 5 min at 4 °C, washed three times, and dried in an air circulation oven at 60 °C, until constant mass. Also, fresh vegetables (controls) from the local market such as asparagus (*Asparagus officinalis* L.), broccoli (*Brassica oleracea* L.), and spinach (*Spinacia oleracea* L.) were dried in an air circulation oven at 60 °C, until constant mass. The dried materials were ground in a mortar, and the granulometry was standardized in particles smaller than 44 µm.

### Iron solubilized by enzymatic hydrolysis

The dried and ground (250 mg) samples (mycelial or vegetable biomass) were mixed to 15 mL ultrapure water, homogenized in a Vortex agitator, and the pH adjusted to 2 with 5 M HCl. Then, 0.75 mL pepsin (20 g L^−1^), previously prepared in 0.1 M HCl, was added and the mixture kept at 37 °C, at 200 rpm, in an incubator shaker, for an hour. The mixture had pH adjusted to six with 1 M NaHCO_3_, addition of 3.75 mL pancreatin solution (8.6 g L^−1^ biliary extract and 1.4 g L^−1^ pancreatin prepared in 0.1 M NaHCO_3_), pH adjusted again to seven with 1 M NaOH, addition of 5 mL NaCl (120 mM), 5 mL KCl (5 mM), and incubated at 37 °C, at 200 rpm, for an hour. After enzyme digestion, the mixture was centrifuged at 15,300g for 10 min at 4 °C, and the clear supernatant and the precipitate were dried in an air circulation oven at 60 °C until constant mass. After that, the iron content was determined.

### Iron content determination

The dried samples of mycelial or vegetable biomass, or their enzymatic hydrolysates (supernatant or precipitate), were added to HNO_3_ (67%), 1:12 (mass:volume), and each mixture was kept at 22 ± 2 °C for 72 h. Each mixture was heated at 100 °C, and H_2_O_2_ (30%), 1:6 (mass:volume), was added. The volume was adjusted to 10 mL with ultrapure water. The iron content of the samples was determined by flame atomic absorption spectrophotometry (GBC model 932 plus). For iron content calculation, a calibration curve (R^2^ = 0.998) was used with analytical standard, certified and traceable (GQ AA Lot 218410115 ultra-scientific analytical solutions) with 0.01 μg mL^−1^ ion detection limit. In addition, for each batch of analyses, an internal standard was placed without the analytical ion and with the standard of the certified element.

The iron availability (%) was calculated by dividing the solubilized iron from the sample by the total iron content in each sample. For fungi, the total iron transfer from the culture medium to the mycelial biomass was calculated by multiplying the mycelial biomass iron content by the mycelial biomass in each batch.

### Statistical analysis

The experimental design was completely randomized, and the arithmetic averages and standard deviations calculated for four biological replicates. The results were submitted to distribution fitting, using maximum likelihood, a two-way ANOVA, and Tukey's HSD (honestly significant difference) test (*p* ≤ 0.05), utilizing the software XLSTAT version 2020.3 (https://www.xlstat.com/en/) and by Software Statistica 13.3 (StatSoft South America, Quest Software Inc, Ok, USA) Serial Number: JPZ711I235230FA-T. The statistical analysis of all data is available for consultation in the supplementary material 1, 2, and 3. 

## Supplementary information

Supplementary Information 1.

Supplementary Information 2.

Supplementary Information 3.
