# Supplementary material for: Iron biofortification and availability in the mycelial biomass of edible and medicinal basidiomycetes cultivated in sugarcane molasses
Source: Sci Rep. 2020 Jul 30;10:12875. doi: 10.1038/s41598-020-69699-0 (PMC7393360; doi:10.1038/s41598-020-69699-0)

**IRON BIOFORTIFICATION AND AVAILABILITY IN THE MYCELIAL BIOMASS OF EDIBLE AND MEDICINAL BASIDIOMYCETES CULTIVATED IN SUGARCANE MOLASSES**

Simone Schenkel Scheidª, Maria Graciela Iecher Fariaª, Leonardo Garcia Velasquez ^b^, Juliana Silveira do Valleª, Affonso Celso Gonçalves Jr. ^c^, Douglas Cardoso Dragunski^d^, Nelson Barros Colautoª, Giani Andrea Lindeª*

ªUniversidade Paranaense, Graduate Program of Biotechnology Applied to Agriculture, Umuarama, PR, Brazil; ^b^Universidade Paranaense, Professional Master in Medicinal Plants and Herbal Remedies in Basic Care; ^c^West Paraná State University, Laboratory of Environmental Chemistry, Centre of Agricultural Science, Marechal Cândido Rondon, PR, Brazil; ^d^West Paraná State University, Center of Engineering and Exact Sciences, Toledo, PR, Brazil

Biomass all Effects


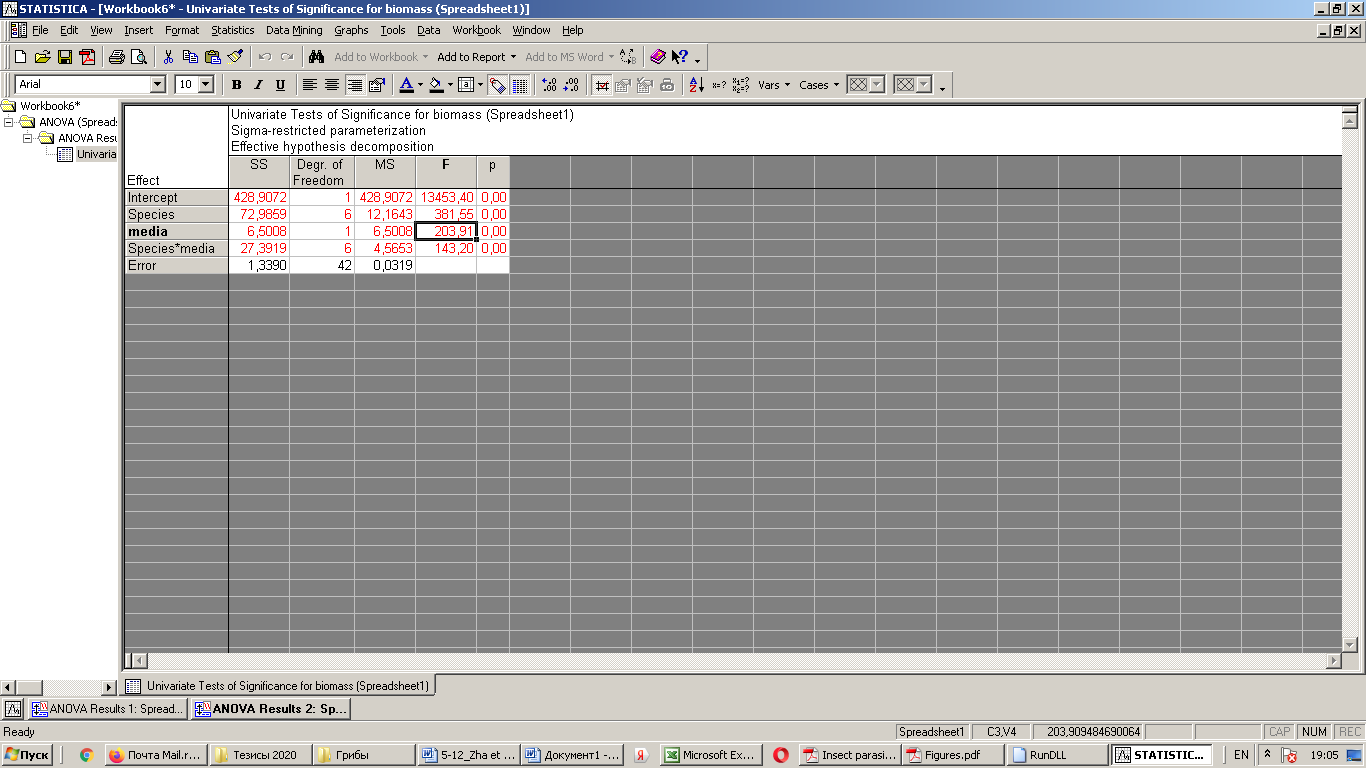


Biomass Tukey post hoc


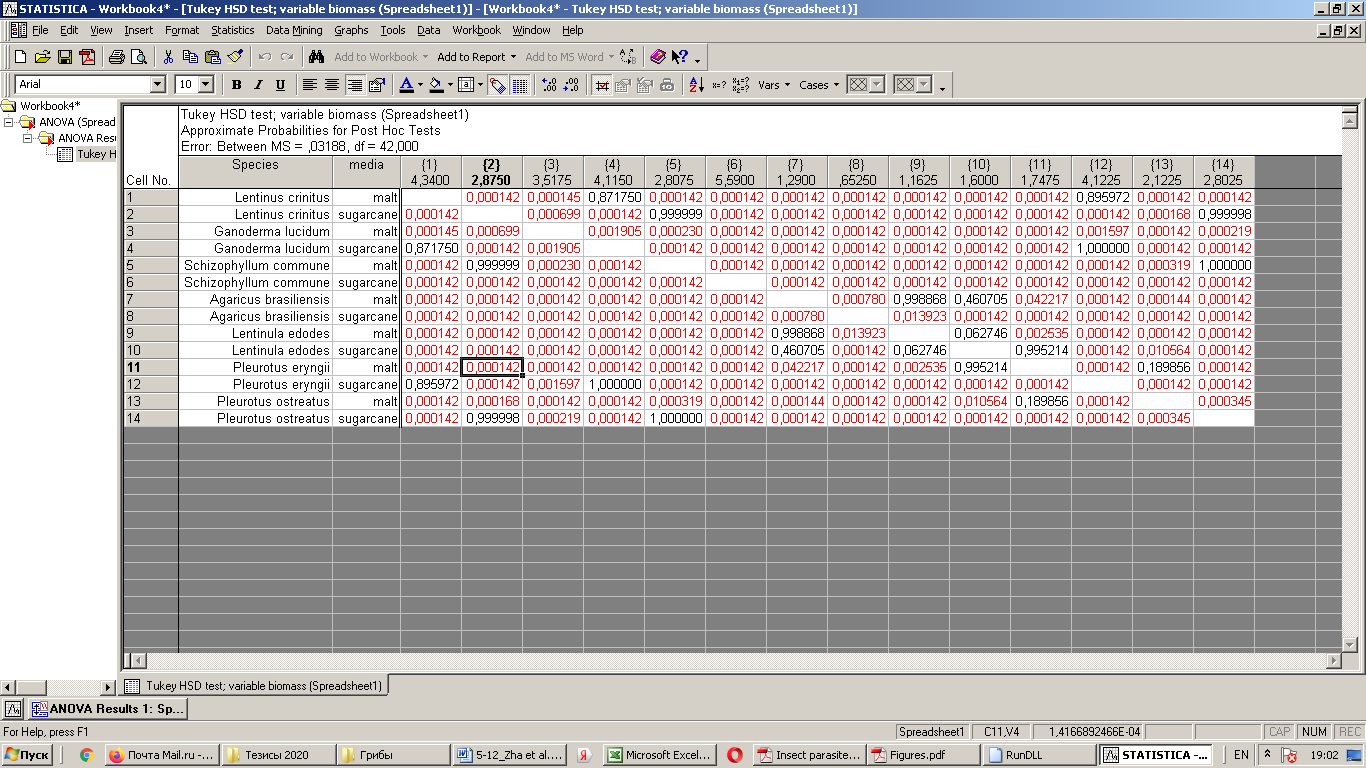


Fe content all effects


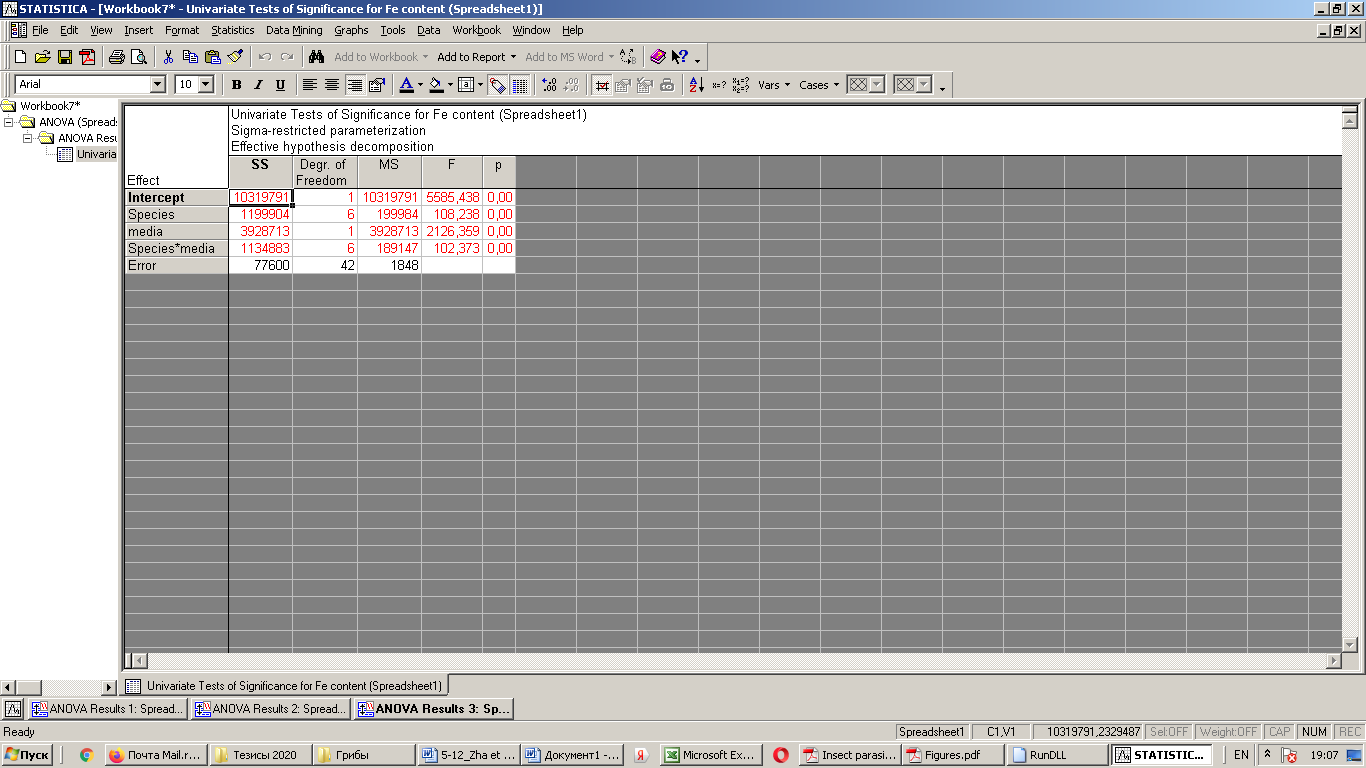


Fe content Tukey post hoc


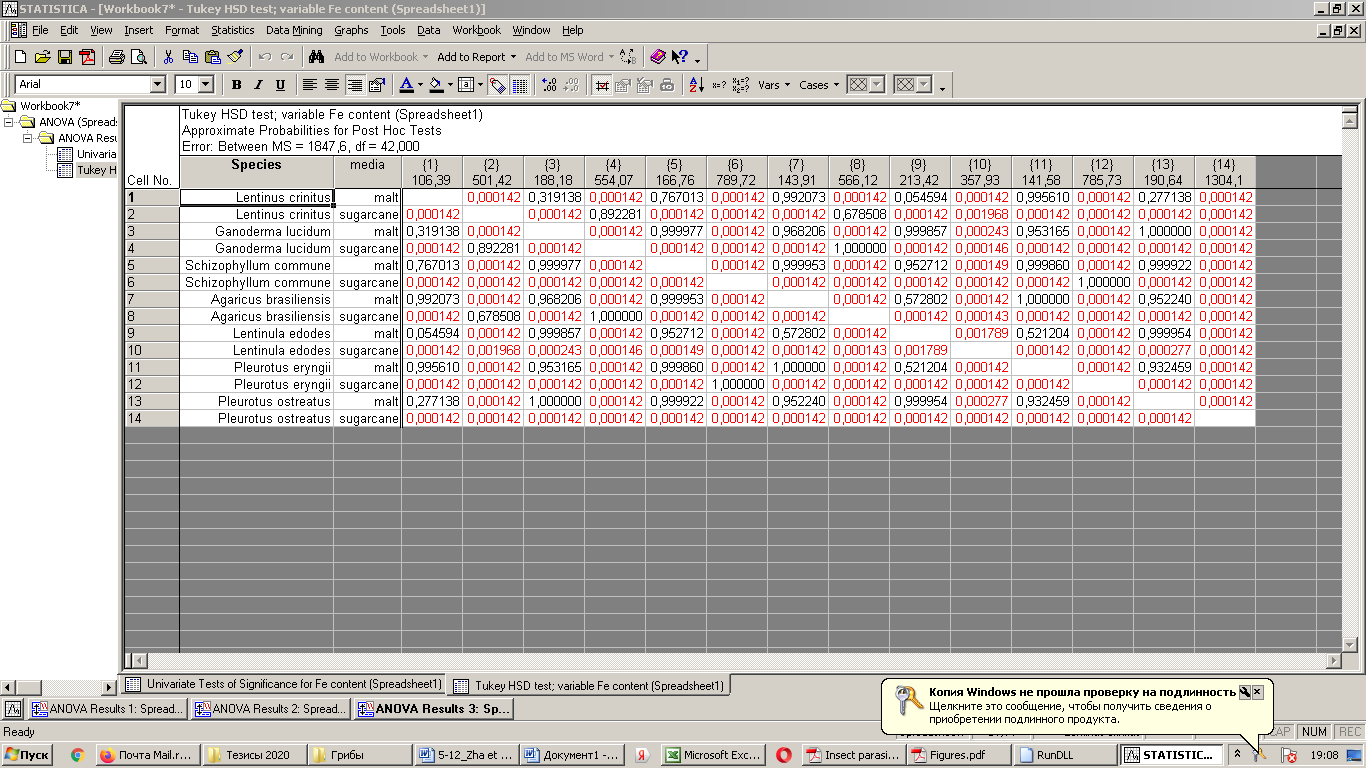


Solub Fe All effects


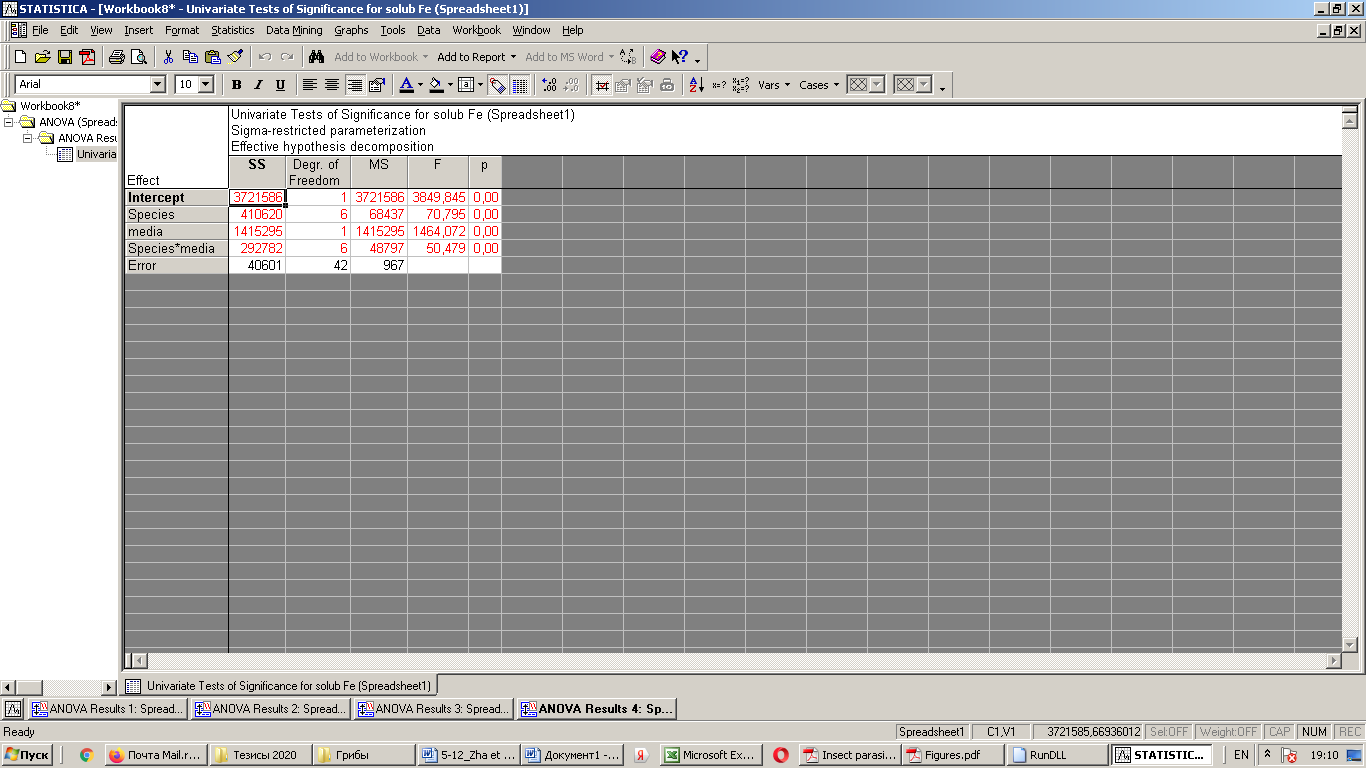


Solub Fe Tukey post hoc


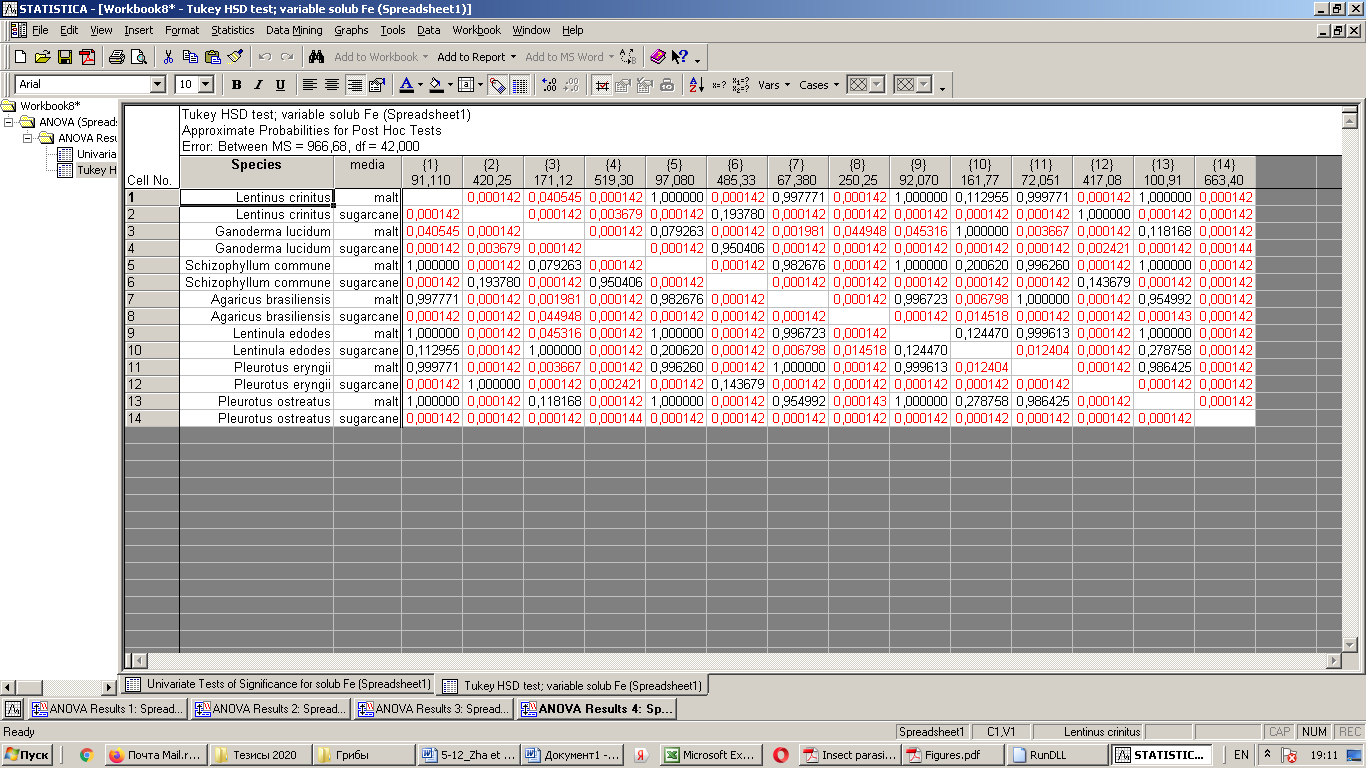


Fe Transfer All effects


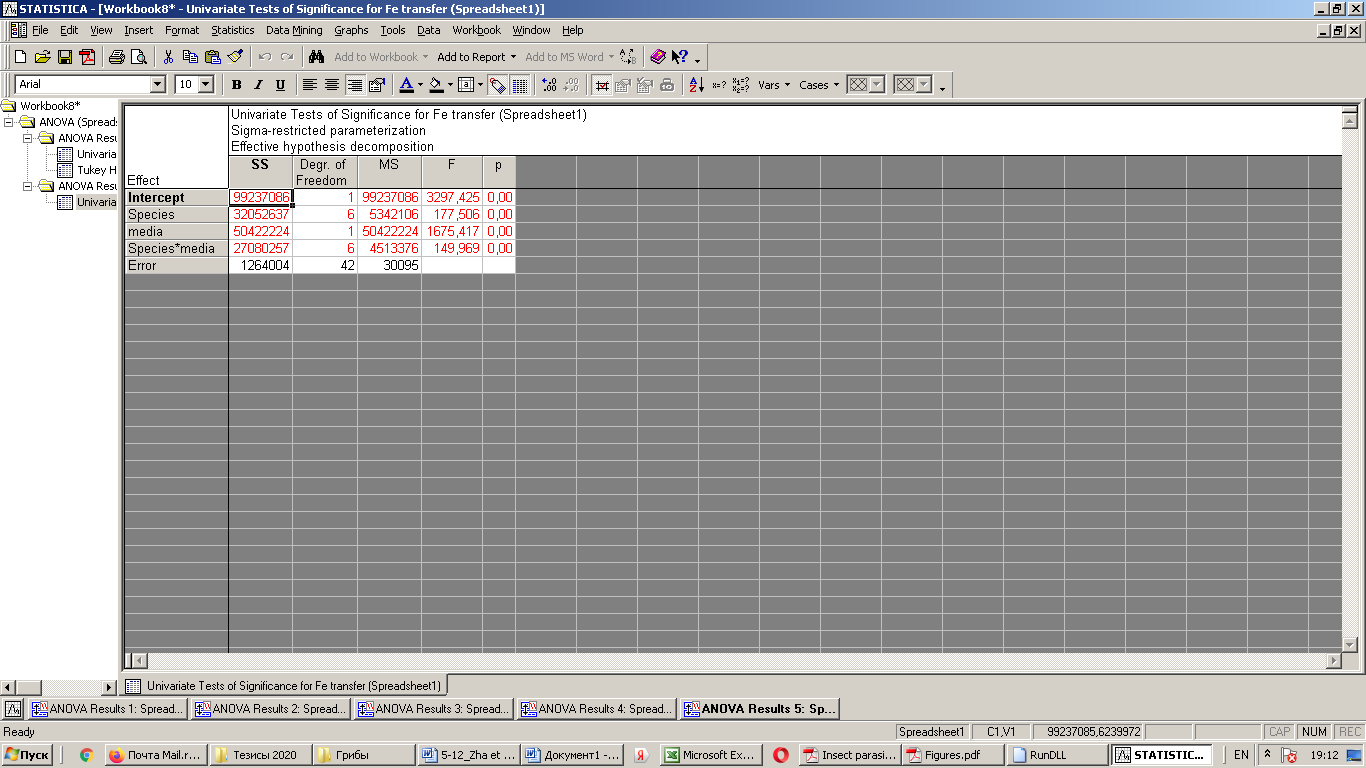


Fe Transfer Tukey post hoc


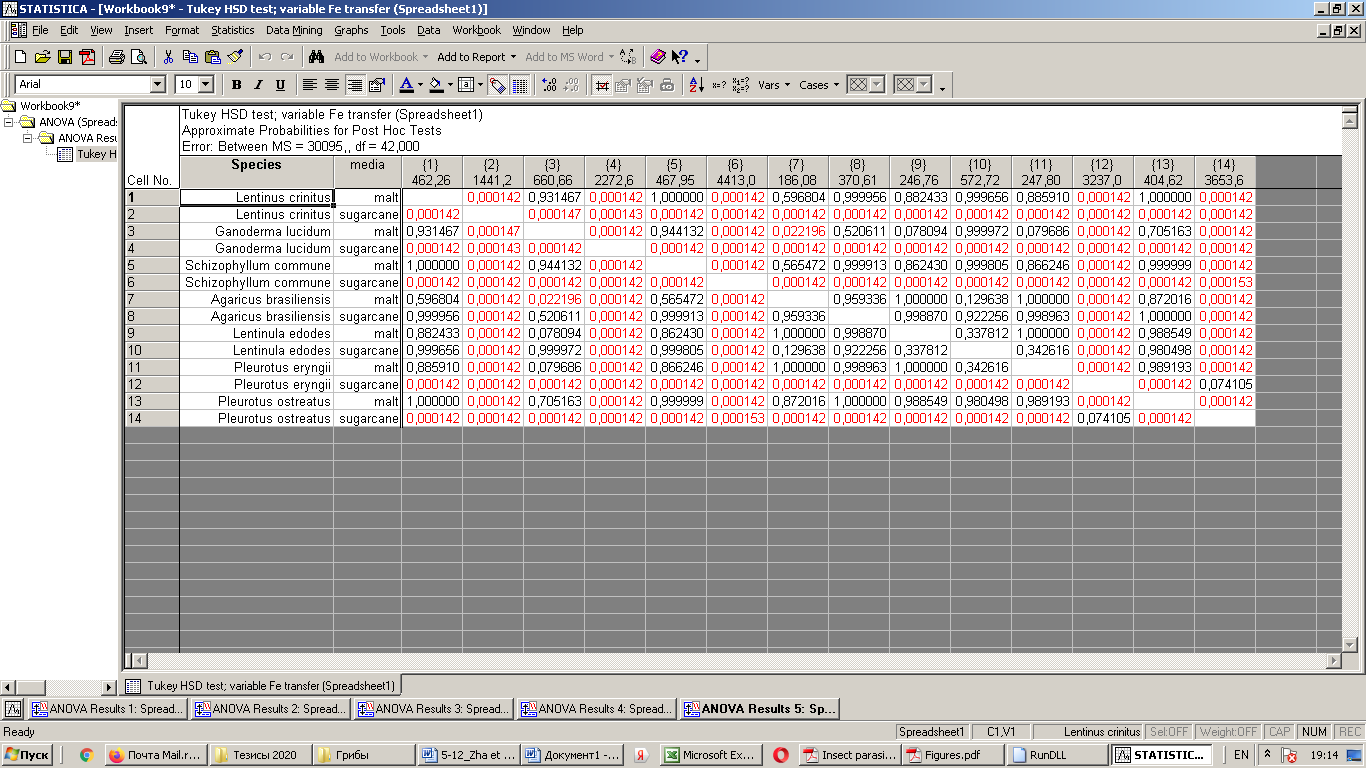


Fe availab All effects


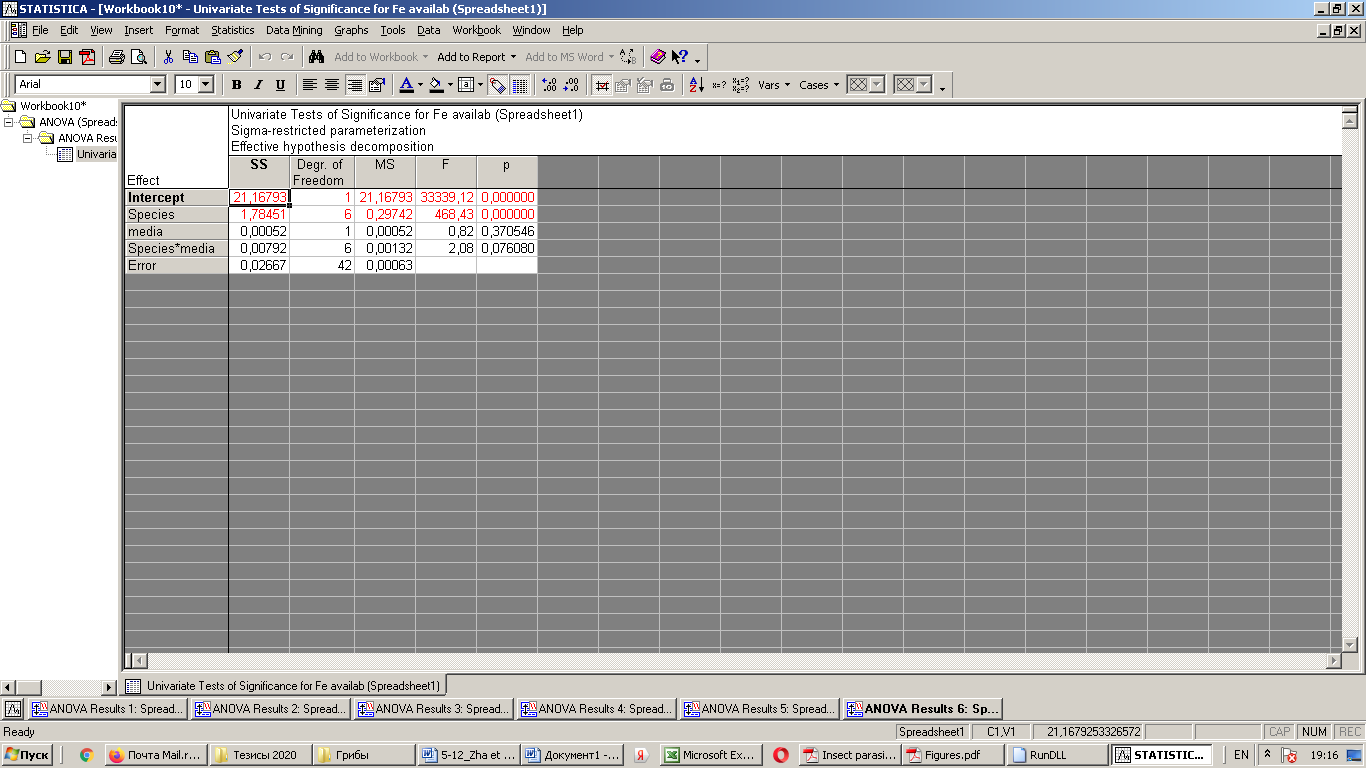


Fe availab Tukey post hoc


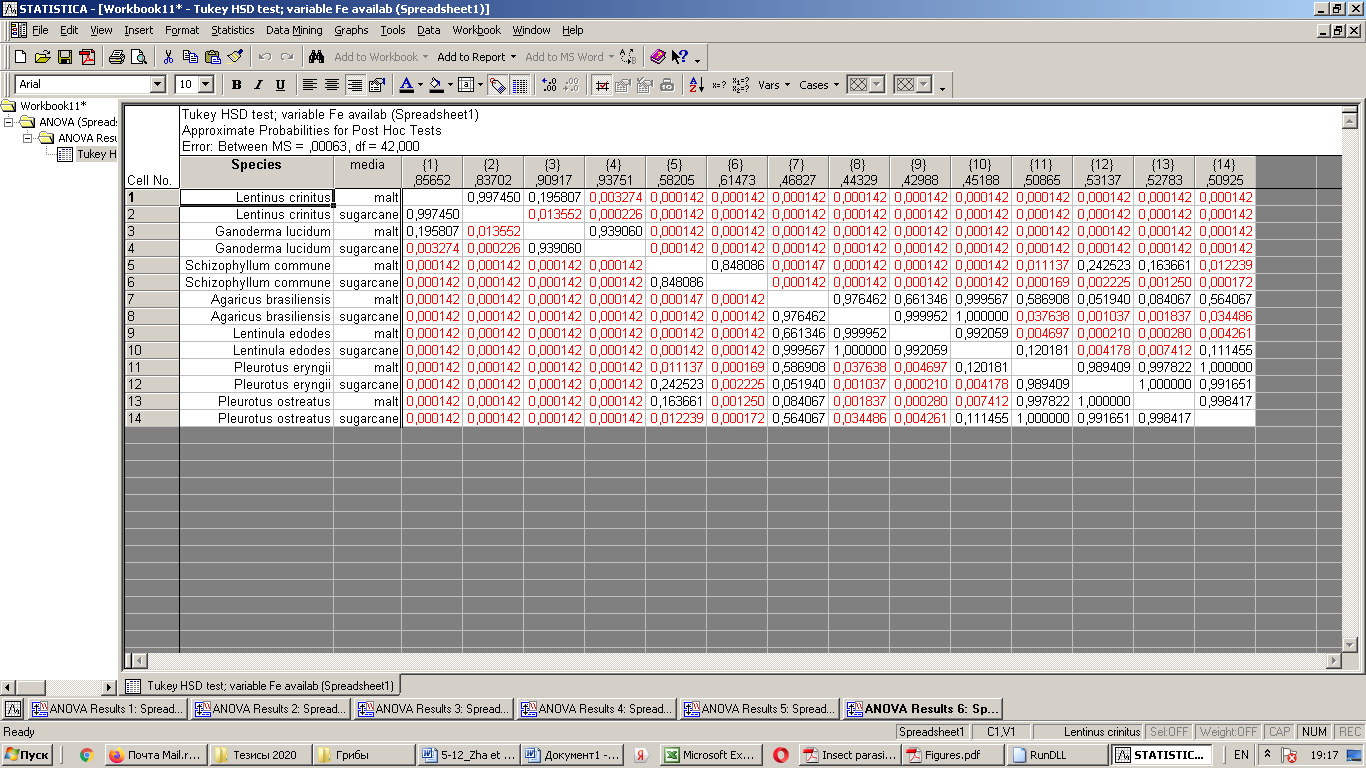


Overall Yeld all effects


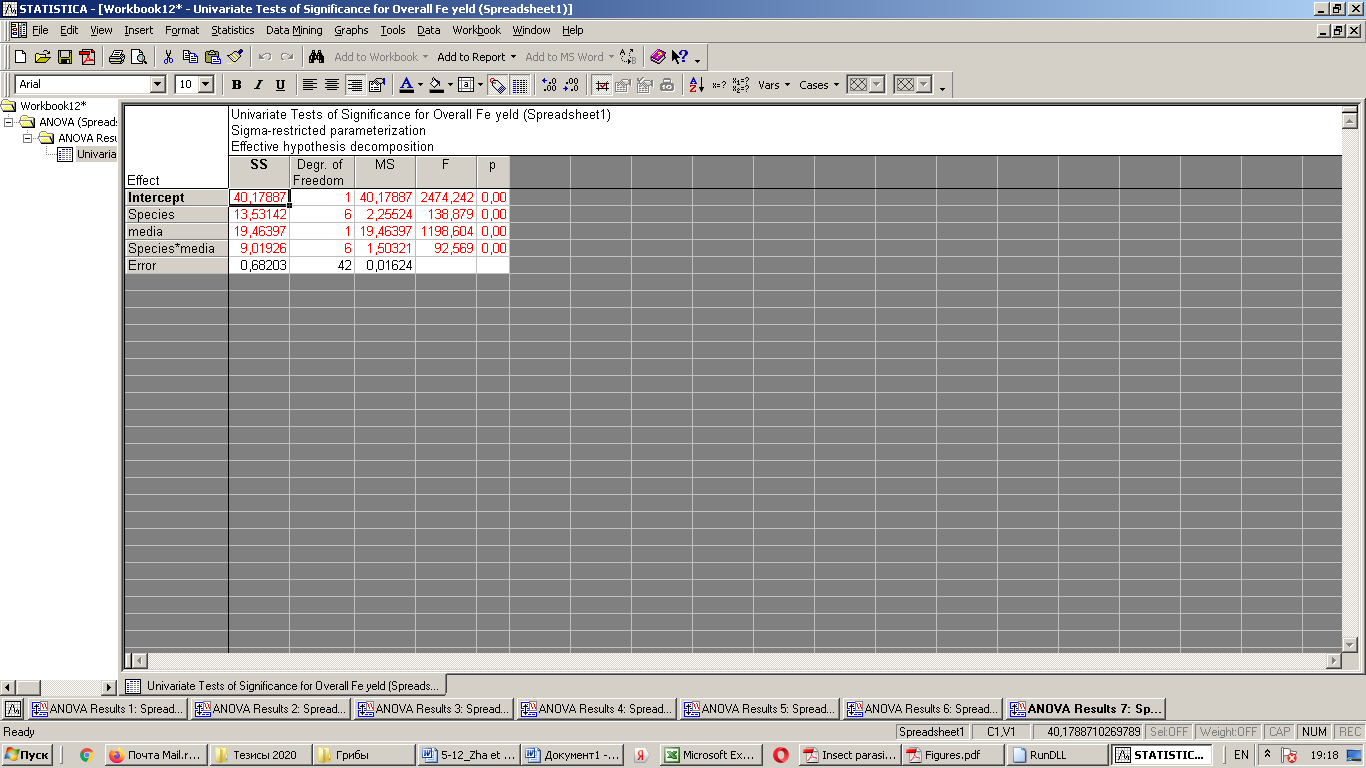


Overall Yeld post hoc


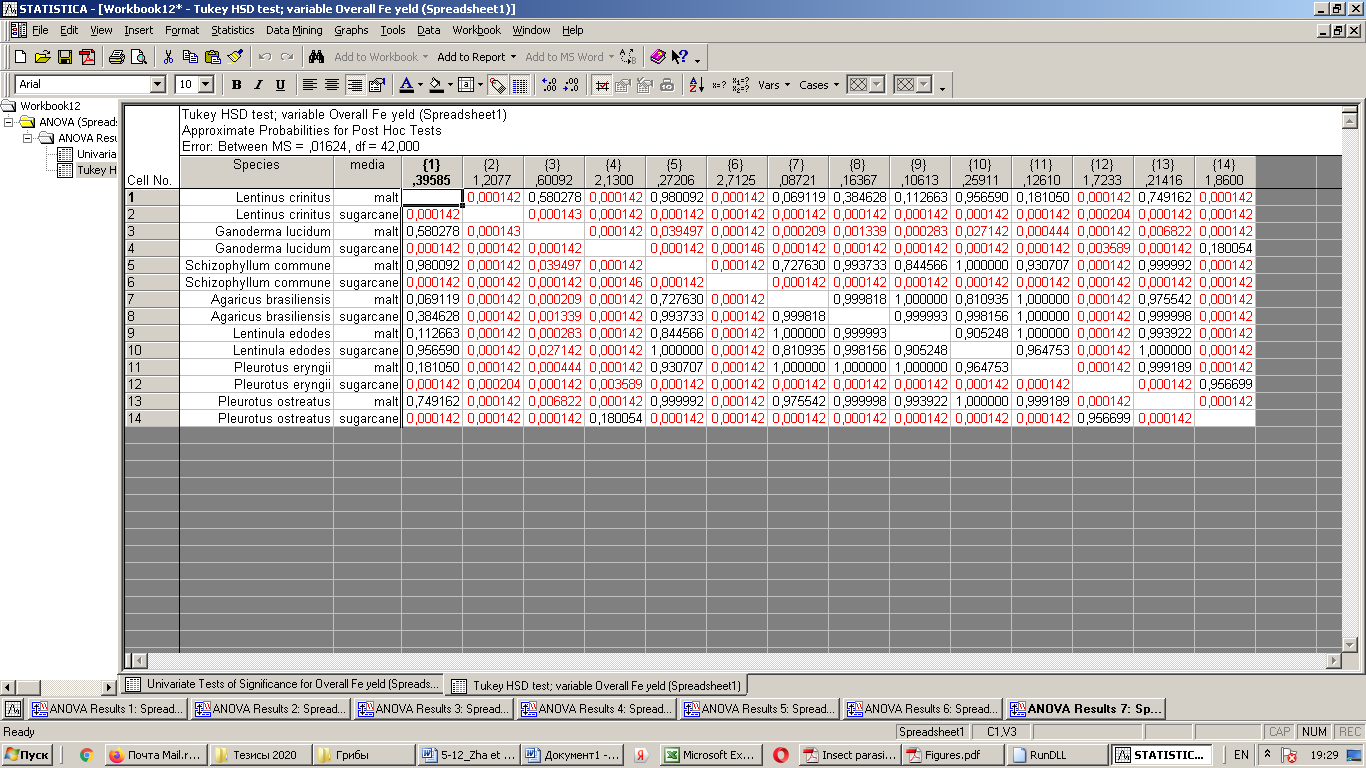

Supplement: Supplementary file 2 — Supplementary Information 2. [file 41598_2020_69699_MOESM2_ESM.doc]
